# Effect and Stability of Poly(Amido Amine)-Induced Biomineralization on Dentinal Tubule Occlusion

**DOI:** 10.3390/ma10040384

**Published:** 2017-04-05

**Authors:** Yuan Gao, Kunneng Liang, Jianshu Li, He Yuan, Hongling Liu, Xiaolei Duan, Jiyao Li

**Affiliations:** 1 State Key Laboratory of Oral Diseases, West China Hospital of Stomatology, Sichuan University, Chengdu 610041, China; gaoyvick@gmail.com (Y.G.); kunnengliang@163.com (K.L.); yuanhe-06@163.com (H.Y.); hongling_l@126.com (H.L.); xingfumo22@163.com (X.D.); 2 Department of Operative Dentistry and Endodontics, West China Hospital of Stomatology, Sichuan University, Chengdu 610041, China; 3 Department of Biomedical Polymers and Artificial Organs, College of Polymer Science and Engineering, Sichuan University, Chengdu 610065, China; jianshu_li@scu.edu.cn

**Keywords:** poly(amido amine), biomineralization, dentine permeability, acid challenge, brushing challenge, stability of dentinal tubule occlusion

## Abstract

In recent years, scientists have developed various biomaterials to remineralize human teeth to treat dentine hypersensitivity. Poly(amido amine) (PAMAM) dendrimers have become a research focus in this field. It has been demonstrated that PAMAM is able to create precipitates both on the surface of and within the dentinal tubules, however, there is little information about its effect on reducing dentine permeability in vitro. This study aimed to evaluate the in vitro effectiveness and stability of the fourth generation amine-terminated PAMAM on dentinal tubule occlusion, especially on dentine permeability. Sodium fluoride (NaF), which has been widely used as a desensitizing agent, is regarded as positive control. Demineralized sensitive dentine samples were coated with PAMAM or sodium fluoride solutions and soaked in artificial saliva (AS) at 37 °C for different periods. Four weeks later, samples in each group were then equally split into two subgroups for testing using a brushing challenge and an acid challenge. Dentine permeability of each specimen was measured before and after each challenge using a fluid filtration system. Dentine morphology and surface deposits were characterized by scanning electron microscope (SEM) and analyzed with Image-Pro Plus software. Data were evaluated through multifactorial ANOVA with repeated measures and pair-wise comparisons at a level of 5%. The results showed that PAMAM and NaF significantly reduced dentine permeability to 25.1% and 20.7%. Both of them created precipitates on dentine surfaces after AS immersion for 28 days. PAMAM-induced biomineralization not only on dentine surfaces, but also deeper in dentinal tubules, significantly reduced dentine permeability. Moreover, PAMAM-induced biomineralization elicited excellent stable occlusion effects after acid challenge. In conclusion, PAMAM demonstrated a strong ability to resist acid and showed great potential to be used in the treatment of dentine hypersensitivity in future.

## 1. Introduction

Dentine hypersensitivity is a common dental problem caused by the exposure of dentinal tubules that allow the movement of intradentinal fluid [1]. Several reviews report that the prevalence of dentine hypersensitivity ranges from 8% to 57% in the general population [2,3]. Brännström et al. proposed the hydrodynamic hypothesis [4], which is widely accepted, suggesting two methods to study desensitize dentine: (a) reducing the ability of intradental nerves to respond to fluid shifts; and (b) minimizing stimulus-evoked fluid shifts in dentinal tubules by decreasing dentine permeability [5,6,7]. Some studies have shown that hypersensitivity occurred on the exposed dentine when most of the tubular orifices were open [8,9], and hypersensitive teeth showed highly significantly increased numbers of tubules per unit area compared with non-sensitive teeth. Tubule diameters were significantly wider, as well [10].

Strategies for managing the condition were remarkably varied. Furthermore, scientific support for various therapies was variable, so it could be a challenge for a practitioner to select an appropriate therapy [11]. Desensitizers should be utilized via both, or at least one, of the two mechanisms of action to relieve dentine hypersensitivity. Sodium fluoride, which is a good remineralization agent, has been widely used as a desensitizer since 1943 [12,13]. Although sodium fluoride reduces dentine permeability within a short period [14], some studies have shown that the mode of action of fluoride occurs through precipitation of calcium fluoride crystals within the tubules [15], and these crystals are not resistant to removal through the action of saliva, brushing, or food substances [16]. What is worse, the chronic ingestion of fluorides in excessive doses during the first years of life may produce enamel alterations, which are characterized for a chalky-white opacity that could eventually affect the complete dental surface, and in some severe cases, may affect the bones [17]. Optimum desensitizing agents for dentine hypersensitivity treatments should reduce dentine permeability and maintain occlusion effects when dentine is subjected to acid challenge and abrasion [18]. In vitro studies have revealed that the risk of dentine hypersensitivity may increase in the presence of dietary acids [19,20]. Acidic substances can remove the smear layer and open dentinal tubules [21,22]. Therefore, further studies should evaluate whether desensitizing agents can effectively occlude dentinal tubules in the oral environment.

In recent years, many researchers have attempted to remineralize demineralized dentine in order to treat the disease. Remineralization is a natural repair process that can only overcome a certain level of acid challenge [23]. Meanwhile, the lack of a nucleation template on the demineralized dentine surface could limit its remineralization effect [24]. Biomimetic remineralization represents a different approach to this problem by attempting to backfill the demineralized dentine with liquid-like amorphous calcium phosphate (ACP) nanoprecursor particles that are stabilized by biomimetic analogs of non-collagenous proteins [25,26,27]. Due to this process, the dentinal tubules would be occluded by newly-formed minerals. It can decrease dentinal fluid movement or dentine permeability, and should decrease dentine hypersensitivity [7].

Poly(amido amine) (PAMAM) dendrimers which are considered to be “artificial proteins” are promising biomaterials for inducing biomimetic dentine remineralization [28,29]. PAMAMs are highly-branched polymers and are characterized by the presence of internal cavities and a large number of reactive end groups with well-defined sizes and shapes [30,31]. PAMAMs are excellent nucleation templates and can rapidly absorb Ca and P ions to cause remineralization, and previous studies have shown PAMAM-induced precipitates in the dentinal tubules with good biocompatibility [32,33,34,35,36,37,38]. Amine-terminated PAMAM (PAMAM-NH_2_) was effective at regenerating minerals on the surfaces of dentine and collagen fibrils [35,37]. However, the ability of PAMAM-NH_2_ to reduce dentine permeability, which can evaluate the degree of dentine sensitivity in vitro, has been poorly described. Meanwhile, the dentine occlusion stability of PAMAM-NH_2_ comparing with sodium fluoride (NaF) in a simulated oral environment has also been rarely reported. Hence, this study used dentine permeability and micrographs to evaluate the effectiveness of the biomimetic remineralization agent PAMAM-NH_2_ on dentinal tubule occlusion at different time periods. Moreover, we selected a brushing challenge and an acid challenge as post-treatments to evaluate the stability of the dentinal tubule occlusion. Dentine permeability was determined by a fluid filtration system. Micrographs were obtained using a scanning electron microscope (SEM) before and after each treatment was administered. The micrographs were then analyzed with ImagePro Plus (version 6, SPSS Inc., Chicago, IL, USA).

## 2. Results

### 2.1. Dentinal Tubule Occlusion at Different Time Periods

#### 2.1.1. Dentine Permeability Measurements

The dentine permeability results obtained in this study were expressed as percentages of maximum permeability, considered equal to 100% after EDTA etching. Table 1 shows the dentine permeability of the three groups at different times.

As time goes on, both PAMAM and NaF significantly reduced dentine permeability. PAMAM reduced permeability more slowly than NaF did. On day 28, the effects of PAMAM and NaF on dentine permeability did not significantly differ (*p* > 0.1). Both PAMAM and NaF groups at 28 days had lower permeability than the control group (*p* < 0.05).

#### 2.1.2. SEM Evaluation

After the EDTA immersion, the dentine surfaces were free of smear layer and smear plugs, and nearly all of the dentinal tubules were completely open.

**Figure 1:**
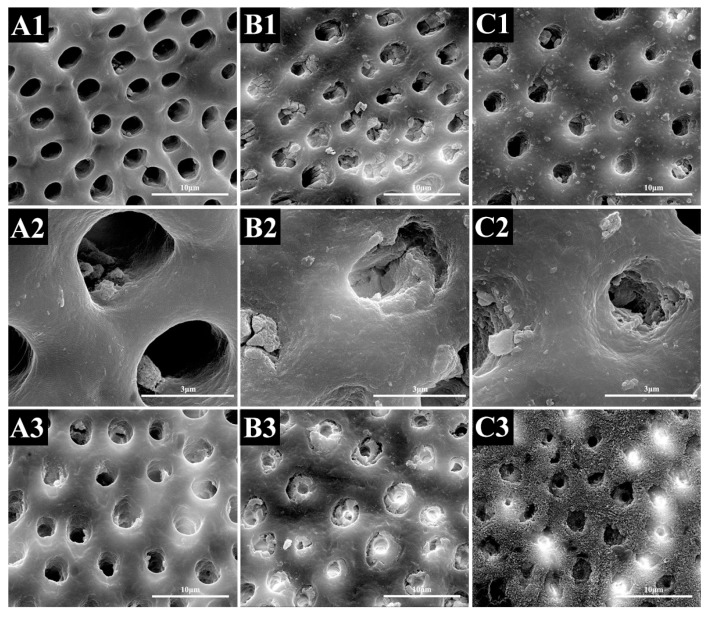

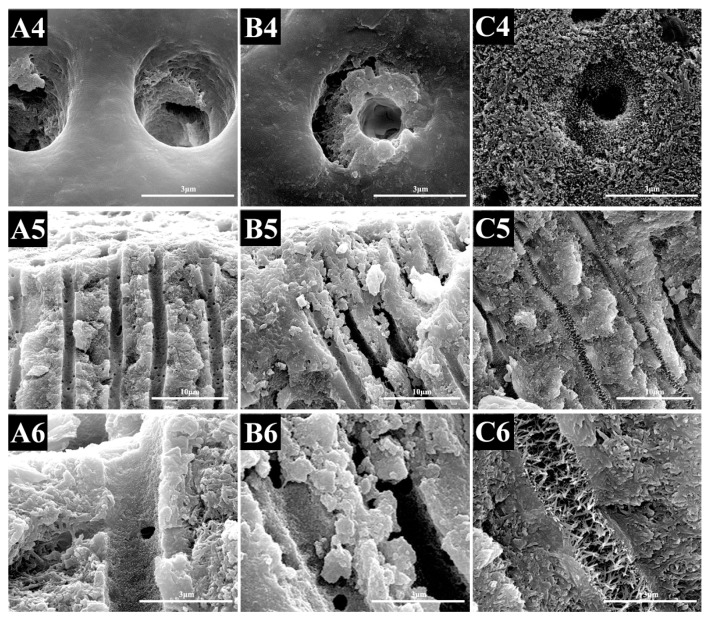
Representative SEM-micrographs of the demineralized dentine surface perpendicular to the tubule axis after seven days (1–2) or 28 days (3–4) of artificial saliva regimen and dentine subsurface cross-section parallel to tubule axis (5–6) after 28 days of artificial saliva regimen: (**A**) control group, (**B**) NaF group and (**C**) PAMAM group. The first, third, and fifth rows are lower magnification (10,000×), and the second, fourth, and sixth rows are higher magnification (40,000×). (**B2**,**C2**,**B4**,**C4**) showed minerals precipitated in dentine markedly. (**C6**) showed that needle-like minerals precipitated in tubules.

The treatments modified the morphological characteristics of the dentine surfaces. The SEM image showed that NaF treatment induced remineralization more quickly than PAMAM did when the specimens were stored in AS. On day 28, there were a few small minerals deposited within the dentinal tubules in the control group, and the dentinal tubules were still completely open. Meanwhile, PAMAM and NaF elicited almost the same effect on tubule occlusion. The precipitates reduced the diameter of the dentinal tubules. However, SEM micrographs of the longitudinal section showed that, in NaF groups, minerals precipitated within the tubules only several microns beneath the surface, while the tubules in PAMAM groups were full of needle-like crystals (Figure 1).

#### 2.1.3. SEM Image Analysis Combines with Image-Pro Plus

**Figure 2:**
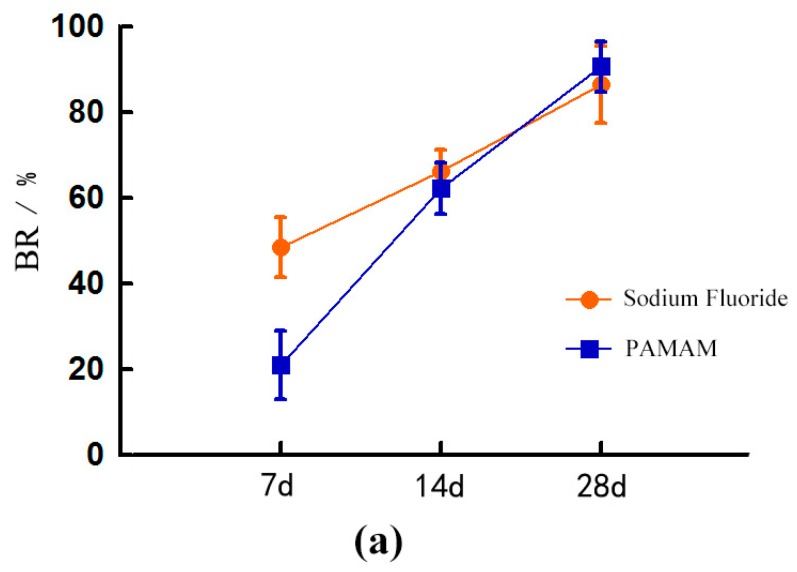

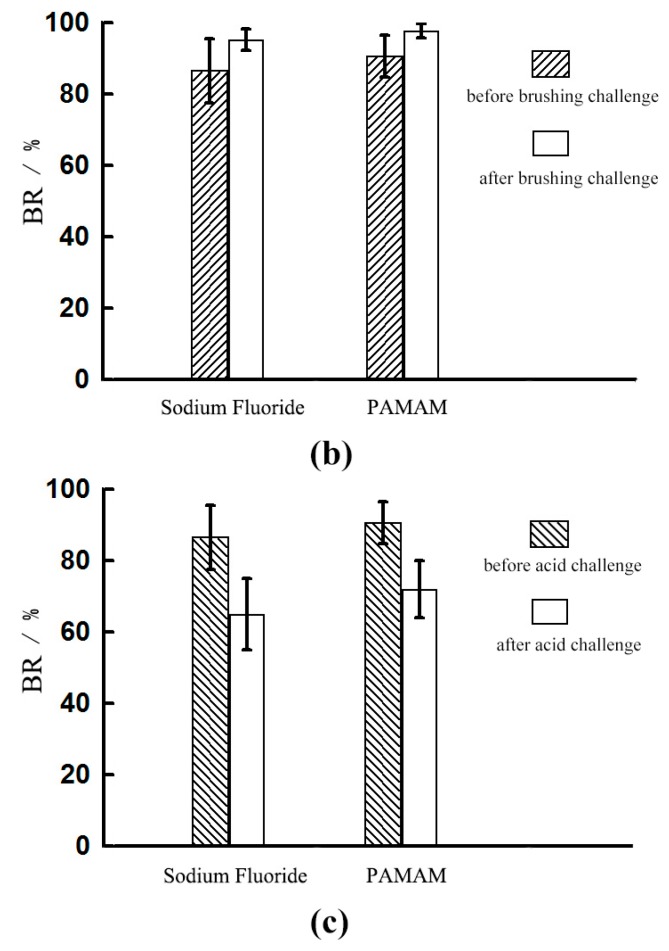
Blocking rate (mean ± SD) of different treated dentine: (**a**) after artificial saliva immersion for 7, 14 and 28 days; (**b**) before and after brushing challenge for 2 min and (**c**) before and after 6% citric acid challenge for 1 min. After 28 days of artificial saliva immersion, PAMAM can have the same effect as NaF. No difference can be found between the two groups after the brushing or acid challenge.

Over time, the blocking rate (BR) of the dentine tubules in the NaF group and the PAMAM group increased significantly. By contrast, the BR of the NaF group was significantly higher than that of the PAMAM group at the seventh day (*p* < 0.05). BR of the NaF group was slightly higher than that in the PAMAM group, but the difference was not statistically significant between the two groups at day 14 and day 28 (*p* > 0.1) (Figure 2).

**Figure 3:**
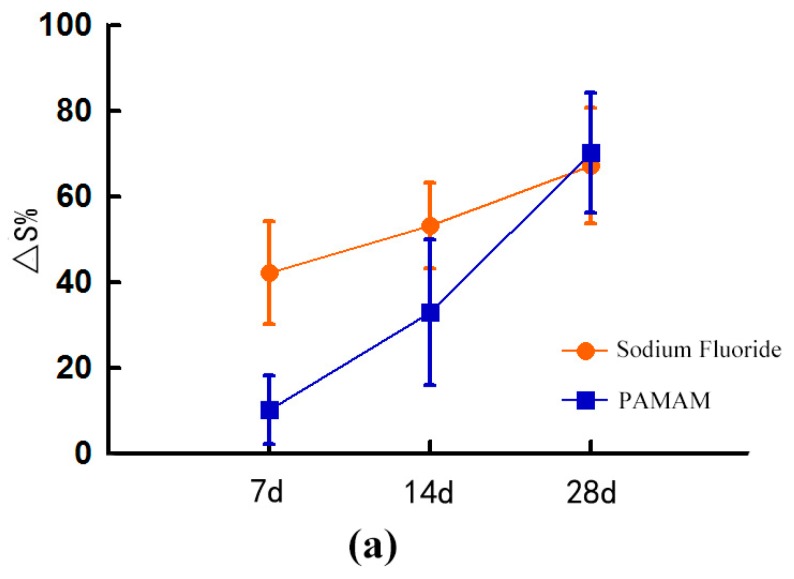

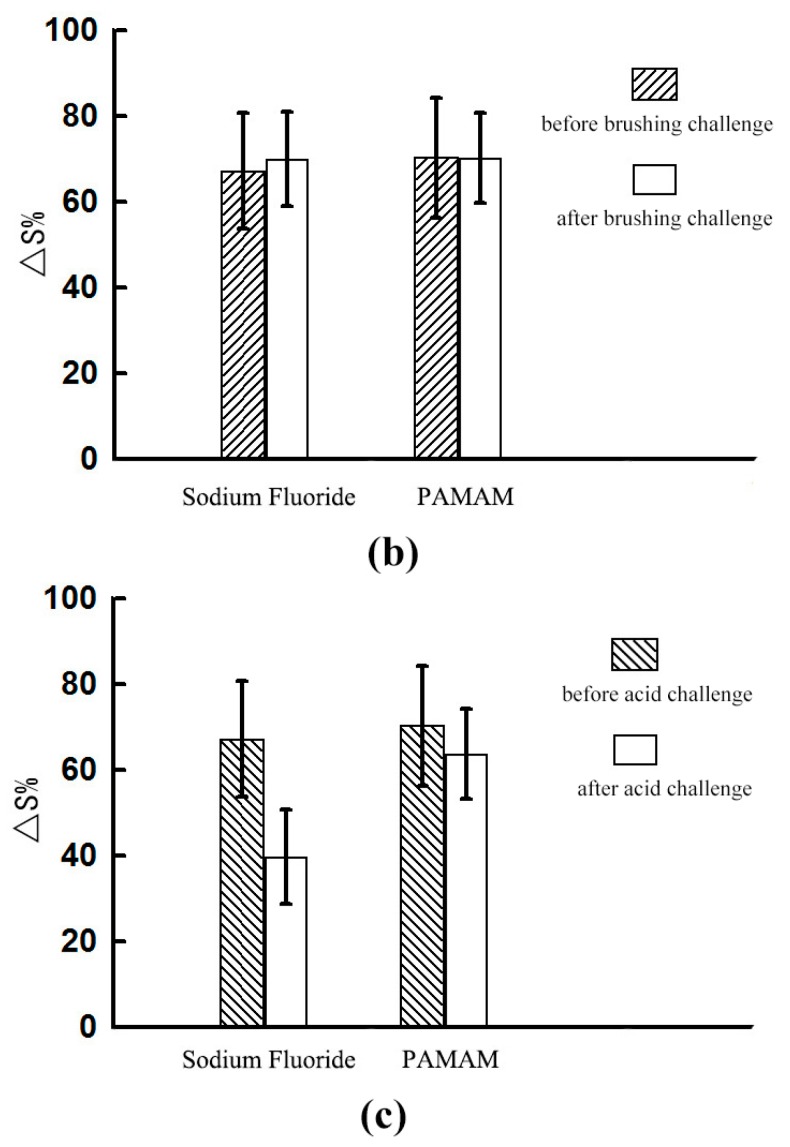
Area change rate (mean ± SD) of different treated dentine: (**a**) after artificial saliva immersion for 7, 14 and 28 days; (**b**) before and after brushing challenge for 2 min and (**c**) before and after 6% citric acid challenge for 1 min. After 28 days of artificial saliva immersion, PAMAM can have the same effect as NaF. Moreover, the PAMAM group demonstrated a stronger ability to resist acid than the NaF group did. However, no difference can be found between the two groups after the brushing challenge.

The Area change rate (∆S%) of the dentinal tubule of the NaF group and the PAMAM group increased. On day 7, ∆S% of the NaF group was significantly higher than that of the PAMAM group (*p* < 0.05). On day 14, ∆S% of the NaF group was still significantly higher than that of the PAMAM group (*p* < 0.05). On day 28, ∆S% of the PAMAM group was slightly higher than that of the SF group, although the difference was no longer significant (*p* > 0.1) (Figure 3).

### 2.2. Dentinal Tubule Occlusion after Brushing Challenge

#### 2.2.1. Dentine Permeability Measurements

After the brushing challenge, the permeability of the distilled water (DIW) group was significantly reduced. By contrast, the permeability of PAMAM and sodium fluoride groups did not significantly differ (*p* > 0.1) (Table 2).

#### 2.2.2. SEM Evaluation

**Figure 4:**
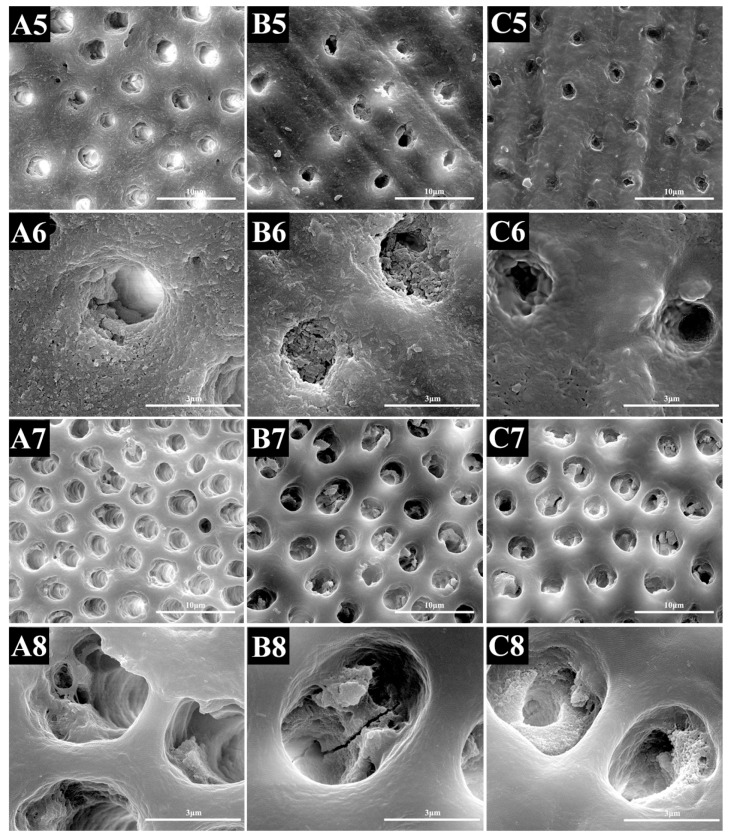
Representative SEM-micrographs of demineralized dentine surface perpendicular to the tubule axis after brushing challenge and acid challenge: (**A**) control group; (**B**) NaF group and (**C**) PAMAM group. The upper two rows are after brushing challenge, and the lower two rows are after acid challenge. The first and third rows are lower magnification (10,000×), and the second and fourth rows are higher magnification (40,000×). (**B6**,**C6**) showed a smear layer on the dentine surface. (**B8**,**C8**) showed residual minerals in dentine tubules.

SEM images revealed that debris was retained on the dentine surface of the specimens in all of the groups. At higher magnification, a new smear layer was formed through brushing; as a consequence, many tubule orifices occluded by small deposits were retained (Figure 4).

#### 2.2.3. SEM Image Analysis Combines with Image-Pro Plus

After the brushing challenge was performed, the BRs of the two groups increased, but the values did not significantly differ between the two groups (*p* > 0.1) (Figure 2). The ∆S% increased in the NaF group and decreased slightly in the PAMAM group, but the differences before and after the test and between the two groups were not statistically significant (*p* > 0.1) (Figure 1).

### 2.3. Dentinal Tubule Occlusion after Acid Challenge

#### 2.3.1. Dentine Permeability Measurements

The citric acid (6%) challenge caused a partial increase in dentine permeability despite the different pre-treatments. PAMAM treatment yielded a significantly lower permeability than sodium fluoride treatment did (*p* < 0.05) (Table 2).

#### 2.3.2. SEM Evaluation

In the citric acid challenge, the number of deposits inside the tubule was reduced and the diameter of the tubule was increased. The PAMAM group contained more crystals in the dentinal tubules (Figure 4).

#### 2.3.3. SEM Image Analysis Combines with Image-Pro Plus

In the acid test, the BRs of the two groups were significantly decreased, but the values did not significantly differ between the two groups (*p* > 0.1). (Figure 2) The ∆S% of the two groups decreased, and the decrease in the NaF group before etching was statistically significant (*p* < 0.05). The PAMAM group showed non-significant difference before and after the test (*p* > 0.1) (Figure 3).

## 3. Discussion

Dentine is a tubular structure composed of hydroxyapatite, organic matrix, and water [39]. Dentine may experience short and severe pain known as hypersensitivity, often caused by acid corrosion, wear, or abrasion. This condition is effectively treated by closing the dentine tubules [40,41]. Most current household or professional desensitizers used to treat dentine hypersensitivity offer temporary relief but often lead to relapse. The most commonly used desensitizer is fluoride, but the mode of action is an inorganic reaction, the induced crystals are not resistant to removal through the action of saliva, brushing, or food substances [16]. Some studies have proven this desensitizer-induced decrease in permeability can be reversed by exposing dentine to acid and saliva [42,43]. Moreover, it causes fluorosis, triggers skeletal fluorosis, and induces other health problems if used excessively [17,44]. Considering these limitations, the present study attempted to introduce biomimetic mineralization into the treatment of dentine hypersensitivity. It determined that both PAMAM and NaF could significantly reduce dentine permeability. Moreover, PAMAM-induced biomineralization elicited an excellent stable occlusion effect after acid challenge.

A nucleation template is the core of the biomineralization process, which can promote the remineralization effect [24]. In recent years, the remineralization of demineralized dentine with biomimetic templates has been of great interest in the fields of material science and stomatology. Some studies have found that PAMAM, a class of mono-dispersed polymeric nanomaterials with plenty of branches radiating from one central core, which possesses a highly-ordered architecture and many calcium coordination sites, can be adsorbed by the positively-charged region of the Ca^2+^ on the surface of hydroxyapatite [30]. This is important to orally fulfill the remineralization function in a fluid-flowing environment [35]. It can also attract free calcium phosphate on the crystal surface; thus, HA crystal growth is promoted along the long axis of the HA crystal. PAMAM dendrimer has been widely investigated in the crystallization process of HA in recent years. The size and shape of HA could be regulated by PAMAM dendrimer with different surface groups, generations, and concentrations [45,46]. Liang et al. found that PAMAM can serve as a mineralization template and induce dentine remineralization with superior depth, good acid resistance, and good biocompatibility [35,37]. Indeed, PAMAM was demonstrated to be an excellent nucleation template for dentine [47].

Previous studies have provided a theoretical basis for the use of PAMAM in desensitization therapy [35,48]. This study establishes an experimental basis for the clinical application of PAMAM by combining the comprehensive analysis of dentine permeability test results and SEM analysis. Dentine permeability was determined to evaluate the degree of dentine sensitivity in vitro. We calculated the blocking rate (BR) by determining the number of the closed tubules, but this parameter could not completely reflect the actual number of closed tubules. By comparison, the change rate of the tubular area was calculated by using the IPP software. The results were more precise and the interference of the external factors could be eliminated by the control group.

During the initial saliva immersion for 14 days, from the result of dentine permeability and SEM analysis, the rate of fluoride-induced mineralization was significantly faster than that of PAMAM. Since sodium fluoride induced mineral deposition via an inorganic reaction with calcium fluoride, the reaction rate was faster than that of organic reactions induced by PAMAM biomimetic mineralization. After 28 days’ immersion, the reaction rate of PAMAM improved, and the dentine permeability became consistent with that of the NaF group. Both PAMAM and NaF groups had lower permeability after 28 days than the control group. SEM micrographs of the longitudinal section revealed the difference between these two modes: the NaF group precipitated minerals within the tubules only several microns beneath the surface, while the tubules in the PAMAM group were full of needle-like crystals. For the control, due to the lack of a nucleation template, all dentinal tubules were open with few mineral deposits. The remineralization rate is important to the depth of occlusion. If the remineralization is too fast, the dentinal tubules will be closed rapidly by the precipitated minerals on the surface, which will prevent Ca and P ions from getting deeper into the dentinal tubules, leading to a poor effect of occlusion [35].

The difference in remineralization can also be seen from the stability test. The brushing challenge was performed to simulate normal brushing and to evaluate the mechanical durability of the tubular seal. The change of dentine permeability showed that the wear resistance of the two groups subjected to NaF and PAMAM treatments did not significantly differ; even the permeability of the control group was significantly reduced. However, the images showed a smear layer on the surface of the treated dentine, and this finding affected the assessment of blocking rate and area change rate. One study used a SEM and observed that different desensitizing toothpastes can obliterate dentinal tubules [49]. It also affirmed that brushing dentine surfaces with toothpastes containing desensitizing products create smear layers or smear plugs that can reduce dentine fluid flow. Therefore, the abrasive resistance of the sealing substance should be further evaluated, such as by means of a nanoscratch experiment.

Citric acid was used to simulate the effects of daily food and oral microorganisms on dentinal tubules to evaluate the durability of the chemical effect of the seal. It has been reported that acidic substances can remove the smear layer and open dentinal tubules [21]. It was found that the number of deposits inside the tubule was reduced and the diameter of the tubule was increased, especially in the control group, while the PAMAM group contained more crystals in the tubules. The results revealed that PAMAM-induced tubular closure protected the teeth against acid more strongly than NaF did. To explain this, we observed that the PAMAM-treated teeth contained significantly deeper closed tubules than the NaF-treated teeth, as shown in the longitudinal section of the SEM micrographs. This phenomenon provided a rapid action to the fluoride treatment, while leading to a lack of stability. The data analysis of SEM micrographs revealed the corresponding conclusion. A previous study revealed that after the citric acid challenge, the dentine permeability of some products increased to 50%–65%, while PAMAM increased to about 30% in the present study [40]. However, in that previous study, they did the acid challenge right after the application of the product, while we immersed the specimens in AS for 28 days before the acid challenge. This would allow the PAMAM-induced biomineralization to penetrate more deeply into dentinal tubules, and give PAMAM greater resistance to acid.

The fluid filtration system is a common device to measure the dentine permeability. However, there is still room for improvement. A previous study used digital flow meter to reduce the reading errors caused by human factors [50]. Some studies used 20 cm H_2_O instead of 703.1 cm H_2_O to simulate the pulpal pressure in the oral condition according to their research need [40,51].

Given the complexities of the oral environment, the pH-cycling experiment may make the acid challenge closer to the real oral condition. Moreover, acid is not the only challenge, oral bacteria and biofilms would be another challenge to the desensitizer. Further animal experiments and clinical trials are needed to analyze and determine the validity of PAMAM, and provide a basis for clinical practice. For example, it could be combined with some rapid-onset medicines to achieve better therapeutic effects.

## 4. Materials and Methods

### 4.1. Dentine Sample Preparation

Human third molars were extracted and collected with informed consent in accordance with a protocol approved by the Ethics Committee of the West China Hospital of Stomatology, Sichuan University (WCHSIRB-D-2015-074). The teeth were cleaned thoroughly and stored in 0.5% thymol at 4 °C within a month prior to their use.

**Figure 5:**
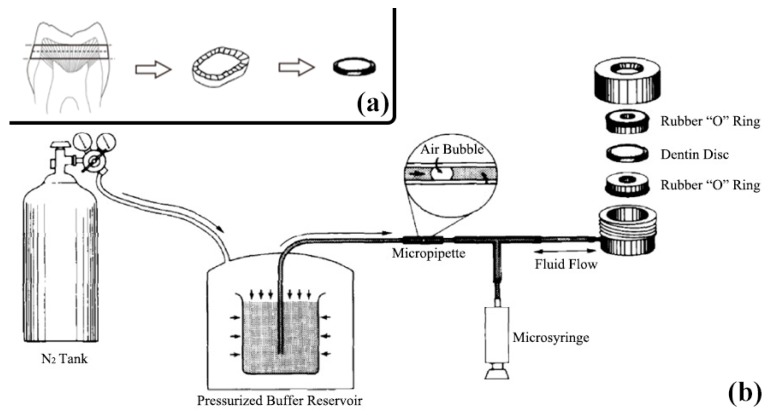
Schematic illustration showing how to prepare dentine disks (**a**) and how dentine permeability was measured by the fluid filtration system (**b**). Each dentine disk was placed in a device that allowed the standardization of exposed dentine area for filtration of deionized water by using pairs of rubber “O” rings. Then hydrostatic pressure of 10 psi was applied and the rate of fluid movement across the disk was measured by following the progress of a small air bubble in a micropipette with a millimeter scale.

Sixty dentine disks, with thickness of approximately 1.0 mm, were cut perpendicular to the long axis of the tooth above the cemento-enamel junction by using a low-speed water-cooled diamond saw. Each disk was carefully prepared and inspected to ensure that they were free of coronal enamel or pulpal exposures (Figure 5a).

After the specimens were prepared, the occlusal surface of each disk was sanded with 800-grit silicon carbide paper for 30 s, then the specimens were immersed in 0.5 M ethylene diamine tetraacetic acid (EDTA) solution (pH 7.4) for 2 min in order to make demineralized sensitive dentine specimens. The samples were ultrasonically cleaned for 10 min and stored at 4 °C in phosphate-buffered saline (PBS, pH 7.4) prior to use [40].

### 4.2. Dentine Permeability Measurement

The specimens were equally distributed into three groups (A–C). Each group contained 10 specimens. Group A specimens were immersed in distilled water (DIW) for 30 min. Group B specimens were immersed in 1000 ppm sodium fluoride solution (NaF) for 30 min. Group C specimens were immersed in 1000 ppm G4-PAMAM-NH2 solution (PAMAM) for 30 min.

The etched disks were rinsed and kept wet to evaluate their maximum permeability, which was set as 100%. The specimens were then stored in artificial saliva (AS) at 37 °C for 28 days. Afterward, permeability was determined on days 7, 14 and 28. The AS solution (pH 7.0) containing 1.5 mM CaCl_2_, 0.9 mM KH_2_PO4, 130 mM KCl, 1.0 mM NaN_3_, and 20 mM 4-(2-hydroxyethyl)-1-piperazineethanesulfonic acid was placed in a 10 mL centrifuge tube, incubated at 37 °C, and replaced with fresh solution every 24 h [52].

**Figure 6:**
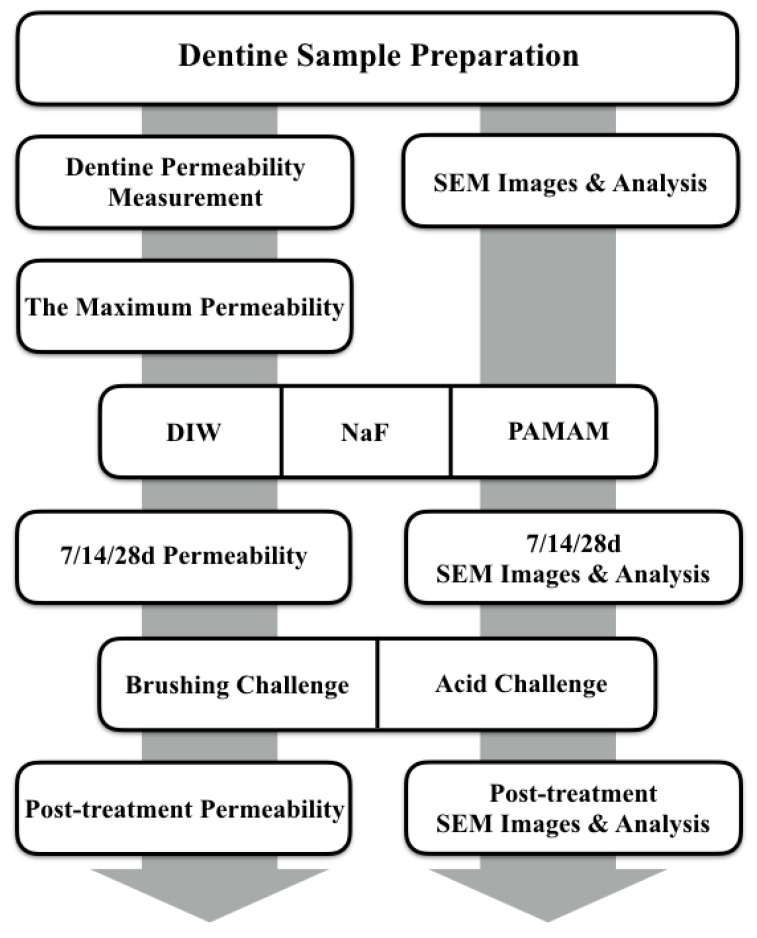
Flow diagram of the experimental design.

The hydraulic conductance (Lp) of the specimens was expressed as the percentage of the maximum EDTA-etched permeability. Subsequently, the specimens were equally distributed into two subgroups and subjected to brushing challenge and acid challenge. Post-challenge permeability was then measured (Figure 6).

The brushing challenge was performed using a toothbrush (SpinBrush Pro, Crest, Guangzhou, Guangdong, China) with bristles of medium hardness, which was applied to the dentine surface at an inclination of approximately 90° with a constant loading of 150 g at 300 strokes/min for 2 min. The brushing load was measured with a top loading balance during brushing. For the acid challenge, specimens were treated with 6 wt % citric acid (pH 1.5) for 1 min and then rinsed with deionized water [40].

Each dentine disk was connected to a water-filled system working at a pressure of 703.1 cm H_2_O. Fluid transudation through the dentine disk was determined by using a fluid filtration system and a modified split-chamber unit. Each dentine disk was held tightly with a pair of rubber “O” rings that provided 0.5 cm^2^ of the available surface area to filter deionized water (Figure 5b).

Fluid flow was measured by following the movement of an air bubble trapped within a 25 mL capacity micro-capillary tube (Microcaps, Fisher Scientific, Atlanta, GA, USA) that was horizontally positioned between the pressure reservoir and the dentine disk. The linear displacement of the air bubble was converted into volume flow (mL/min) [53]. For each specimen, fluid flow across the dentine disks was then transformed into hydraulic conductance (mL/min·cm^2^ cm H_2_O) by dividing the fluid flow (mL/min) by the exposed dentine surface area (cm^2^) and water pressure (cm H_2_O).

The permeability of each specimen was expressed as the percentage (Lp %) of the fluid flow through the EDTA-etched dentine disk of the same specimen. To ensure that a tight seal was provided by the “O” rings and to check the system for leaks, we inserted a polyethylene disk in place of the dentine disk. No filtrate was recorded.

### 4.3. SEM Analysis

**Figure 7:**
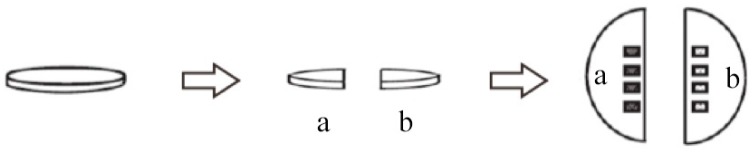
Schematic illustration showing how dentine disks for SEM analysis were created. Using dental pliers, the disks were broken in half to provide control (**a**) and experimental (**b**) sections. After different treatment in each group, micrographs were taken from the central portion of each disk where the tubules were more or less round and each pair of control and experimental images were as close together as possible. Four micrographs of the control and four corresponding experimental images were taken at a constant magnification of 10,000 times.

The specimens for SEM analysis were equally distributed into two groups (B’ and C’). Each group contained 10 specimens. The specimens were broken in half by using dental pliers to provide control (a) and experimental (b) sections (Figure 7). The control halves were treated as those in group A mentioned before, and the experimental halves in group B’ or C’ were treated with NaF or PAMAM solution, respectively [54].

The specimens were then stored in AS at 37 °C for 28 days. Afterward, two specimens were taken out of each group to perform SEM analysis on days 7, 14 and 28. Subsequently, the residual specimens in each group were equally distributed into two subgroups and subjected to the above-mentioned brushing challenge and acid challenge. Then post-treatment SEM analysis was performed (Figure 6).

The specimens were dried in a desiccator and sputter-coated with gold in a vacuum evaporator. The micrographs of the dentine surface were obtained using a SEM. In particular, micrographs were collected from the central portion of each disk, where the tubules were more or less round, and each pair of test and control images was as close as possible. Four micrographs of the control images and four corresponding test images were taken on one film at a constant 10,000× magnification, with a scale bar of 10 µm. Thus, any subsequent processing was the same for all negatives of each disk. Images were analyzed using ImagePro Plus v6.0 for Windows (SPSS Inc., Chicago, IL, USA). Each image was calibrated individually. Only the tubules within the image were measured. The tubules that overlapped the edges of the marked area and confused the data by appearing only in part were not measured [54].

The measurements included in the data were blocking rate (BR) or the ratio of the number of the occluded tubules to the number of all tubules in the image (N).

BR = (1 × M + 0.5 × m)/N

where M is the number of tubules occluded by >50% and m is the number of tubules occluded by <50%.

Area change rate (∆S%) is the ratio of the change in area between subgroups c and d.

$$∆S\%=(S\bar{c}-S\bar{d})/S\bar{c}$$

where S$\bar{x}$ is the unoccluded area of subgroup x.

### 4.4. Statistical Analysis

Statistical analysis was performed with SPSS 16.0 for Windows. The means and standard deviations of Lp % were calculated. Data were reported as the percentage of the original maximum permeability values. The prospective and retrospective power calculation should be done to confirm the sample size is enough. The homogeneity of variance was assessed via Levene’s test. Two-way repeated measure ANOVA was applied to evaluate dentine permeability, level of significance was set at α = 0.05 (two-sided), with treatment as the main effect and treatment time as the repeated measure.

## 5. Conclusions

In the present study, we found that the rate at which PAMAM-induced biomineralization occluded dentinal tubules was slower than the rate for NaF-induced biomineralization. However, after 28 days of treatment, both treatments elicited the same effect. PAMAM exhibited stronger acid resistance than sodium fluoride, which indicates that it achieves a more stable dentinal tubule occlusion. Although the effects need to be validated in animal experiments and clinical trials, we believe that PAMAM has great potential to be used in the treatment of dentine hypersensitivity.

## Figures and Tables

**Table 1:** Dentine permeability of three groups at different time (%, *N* = 10, X ± SD).

Groups	Immediate	7 d	14 d	28 d
**DIW**	98.5 ± 4.5	91.2 ± 3.3 *	88.3 ± 3.0 *	82.6 ± 3.4 *
**NaF**	71.6 ± 6.5 *	40.9 ± 7.2 *	23.3 ± 4.2 *	20.7 ± 3.1
**PAMAM**	90.1 ± 7.4	77.3 ± 8.1 *	57.6 ± 8.5 *	25.1 ± 6.3

* means it has significant difference with other groups, *p* < 0.05.

**Table 2:** Dentine permeability after brushing or acid challenge (%, *N* = 10, X ± SD).

Groups	Abrasive Test	Acid Challenge
**DIW**	58.3 ± 8.8 *^,^**	105.8 ± 9.2 *^,^**
**NaF**	17.3 ± 9.1	61.2 ± 8.7 *^,^**
**PAMAM**	18.6 ± 9.4	33.1 ± 9.0 **

* means it has significant difference between pre- and post-treatment; ** means it has significant difference with other groups.
